# Effect of low- and high-dose methotrexate on wound healing in rats

**DOI:** 10.1590/acb403225

**Published:** 2025-03-10

**Authors:** Abdullah Karasu, Yağmur Kuşcu, Caner Kayikci, Serkan Yildirim, Oğuzhan Kuşcu, Metin Kiliçlioğlu

**Affiliations:** 1 Van Yuzuncu Yil University – Faculty of Veterinary Medicine – Department of Surgery – Van – Türkiye.; 2 Ataturk University – Faculty of Veterinary Medicine – Department of Pathology – Erzurum – Türkiye.; 3 Van Yuzuncu Yil University – Faculty of Medicine – Department of Histology and Embryology – Van – Türkiye.

**Keywords:** Wound Healing, Methotrexate, Rats

## Abstract

**Purpose:**

To investigate the effect of intraperitoneal treatment with low- and high-dose methotrexate (MTX) on wound healing in rats.

**Methods:**

The study sample consisted of 54 healthy rats. Under aseptic conditions, skin wounds were created with two circular full-thickness punch tools, 10 mm in diameter, one on the right and the other one on the left of the dorsal vertebral line. The rats were randomly assigned to one of three main treatment groups. On the 0th day (2 hours before wound creation), 7th day, and 14th day, the control group received 0.3-mL saline, the low-MTX group received 3 mg/kg MTX, and the high-MTX group received 30 mg/kg MTX, all administered intraperitoneally. The wounds were evaluated seven, 14, and 21 days after injury through morphometrical, biochemical, histopathological, and immunohistochemical analyses.

**Results:**

MTX dose-dependently decreased the degree of inflammation and angiogenesis, tissue hydroxyproline level, and HSP70 and tumor necrosis factor-α expression in the early phase of wound healing. It also suppressed epithelialization and collagen 1 expression throughout the wound-healing process.

**Conclusion:**

The wounds treated with high-dose of MTX had statistically delayed wound closure on days 7, 14 and 21 compared to the saline group, while wounds treated with low-dose of MTX only had statistically delayed wound closure on day 14. In addition, weight loss was observed in rats treated with high-dose MTX, which was thought to reflect its toxicity. The dose-dependent adverse effect of MTX on wound healing may be due to its antiproliferative, antifibrotic, anti-inflammatory, and antiangiogenic effects.

## Introduction

A wound disrupts the anatomical integrity and functional continuity of soft tissues. Wound healing is a sequence of cellular and biochemical events that begins with trauma and results in repairing tissues’ anatomical and functional integrity^1^. It comprises sequential, overlapping phases, including inflammation, proliferation, and remodeling^2^. The inflammatory phase is characterized by vascular responses that result in blood coagulation and hemostasis, as well as cellular infiltration, primarily by leukocytes with diverse functions^1^. In addition, various growth factors, e.g., platelet-derived growth factor, epidermal growth factor, and fibroblast growth factor, are secreted by blood platelets. The entry of growth factors into the wound requires the migration of cells involved in wound protection from microbial contamination–monocytes, macrophages, neutrophils, and leukocytes^3^. In the proliferation phase, endothelial cells promote angiogenesis. Fibroblasts produce large quantities of extra-cellular matrix (ECM) to form granulation tissue to repair damaged tissue. Keratinocytes play a crucial role in the healing process by covering wound surfaces to reestablish an epithelial barrier with the outside environment. They produce multiple factors to promote re-epithelialization and stimulate angiogenesis and production of connective tissue matrix^4^. In the final stage of wound healing, maturation, the granulation tissue matures into a scar and the tensile strength of the tissue increases^3^.

Most chemotherapeutic drugs inhibit cellular metabolism, rapid cell division, and angiogenesis^5^. They may also exhibit toxicity against normal cells, such as keratinocytes and fibroblasts, and, therefore, inhibit many critical wound repair pathways. These drugs inhibit DNA, RNA, or protein synthesis, decreasing fibroplasia and neovascularization in wounds^3^^,^ 5. Chemotherapeutic drugs also delay cell migration to the wound, reduce early wound ECM formation and collagen production, disrupt the proliferation of fibroblasts, and prevent wound contraction^5^. Thus, they disrupt the wound-healing process.

Methotrexate (MTX) is an antimetabolite used in cancer treatment that limits the synthesis of the purines and pyrimidines required for nucleic acid synthesis by inhibiting the dihydrofolate reductase enzyme. In addition to its antineoplastic properties, MTX also exhibits anti-inflammatory, antiproliferative, immunosuppressive, and antipsoriatic activity and is still used in human and veterinary medicine for these purposes^6^. In human medicine, this anti-neoplastic agent is used in high-dose regimens, mainly to treat childhood acute lymphoid leukemias, other hematological and trophoblastic malignancies, and other cancers. Although it was originally developed as an anti-neoplastic agent, low-dose MTX has demonstrated high effectiveness in treating immune-mediated disorders, such as rheumatoid arthritis, psoriatic arthritis, and other inflammatory conditions. Despite the new biological agents that have revolutionized the treatment of many of these conditions, MTX remains the preferred drug in many cases^7^. As MTX has cytotoxic and antiproliferative effects, it affects not only cancer cells, but also all rapidly dividing cells, both healthy and malignant^8^.

There is limited evidence in the literature regarding MTX’s effects on wound healing in experimental animals. Few experimental studies investigated MTX’s effects on wound healing, and those conducted in the 1960s and 1980s before modern methodological standards were developed were also limited to macroscopic evaluations^9^. A recently published study reported that there is little information on MTX effects in the wound remodeling process using in-vivo models^10^. The lack of comprehensive research on MTX’s effects on wound healing in the literature encouraged us to conduct this study. This study investigated the effect of low- and high-dose MTX on wound healing in the morphometric, biochemical, histopathological, and immunohistochemical dimensions.

## Methods

### Ethical statement

The study was conducted after the study protocol was approved by the animal experiments local ethical committee of Van Yüzüncü Yil University (Approval no.: 2019/05).

### Animals

For this study, 54 clinically healthy male Wistar Albino rats weighing 200–300 g were used. The animals were periodically weighed before and after the experiment. They were housed under standard environmental conditions (23 ± 1°C, at 55 ± 5% humidity, with a 12-h light/dark cycle). They were fed commercial pellet food and given freshwater ad libitum. Experiments were performed during the light phase of the cycle (between 8:30 and 12 hours). The procedures involving animals and their care adhered to international guidelines and the principles of laboratory animal care.

### Excision wound model

The rats were anesthetized with an intramuscular injection of a mixture of 80 mg/kg of ketamine (Ketamidor, Richter Pharma), and 10 mg/kg of xylazine (Rompun, Bayer). Both sides of the animals’ backs were shaved, and the skin was disinfected with povidone iodine. Under aseptic conditions, skin wounds were created with two circular full-thickness punch tools, 10 mm in diameter, one on the right of the dorsal vertebral line for biochemical analyses and one on the left for histopathological and immunohistochemical analyses.

### Experimental design

The animals were randomly assigned to one of three main treatment groups (n = 18 per group). On the 0^th^ (2 hours before wounds were created), 7^th^, and 14^th^ days, 0.3-mL saline (0.9% NaCl-Polifarma), 3 mg/kg MTX (Methotrexate, Koçak), and 30 mg/kg MTX were administered intraperitoneally to the control group, the low-dose MTX group (low-MTX–mimicking rheumatoid arthritis dose in humans), and the high-dose MTX group (high-MTX–mimicking chemotherapeutic dose in humans), respectively^11^^–^ 13. No application was made to the wounds, and they were left open to secondary intention. Each main group was randomly divided into three subgroups to be sacrificed on the 7^th^, 14^th^, and 21^st^ days14 for histopathological, immunohistochemical, and biochemical analyses (n = 6 per subgroup).

### Measurement of wound area

Digital photographs of the wounds were taken on days 0, 7, 14, and 21 after injury. A single blinded observer performed the analysis of the digital photographs by tracing each wound edge with a computer mouse to calculate the pixel area, which was then converted to square mm using the free image analysis software Image J. Wound area at each time point was additionally expressed as percentage closure of the original wound (Eq. 1):


$$\text{Wound area}=\text{Wound size at baseline}\left({\mathrm{mm}}^{2}\right)\text{- Wound area at a given day}\left({\mathrm{mm}}^{2}\right)/\text{Wound size at baseline}\left({\mathrm{mm}}^{2}\times 100^{15}\right)$$ (1)

### Biochemical evaluation

The tissue samples were excised in wound. Tissues were rinsed in ice-cold phosphate buffered saline (PBS) (pH 7.4) to remove excess blood thoroughly and weighed before homogenization. Tissues minced and homogenized in PBS [tissue weight (g): PBS (mL) volume = 1:9] with the rotor-stator homogenizers on ice. The homogenates were then centrifuged for 15 minutes at 12,000 RPM at 4°C to get the supernatant. Using a hydroxyproline and vascular endothelial growth factor (VEGF) enzyme-linked immunosorbent assay (ELISA) kit (BT LAB, Shanghai, China), the hydroxyproline and VEGF amounts were calculated in nanograms per milliliter of wet tissue by reading the absorbance of the solution on a spectrometer at 450 nm.

### Histopathological evaluation

Tissue samples taken for histopathological examination were fixed in 10% formalin solution for 48 h. As a result of routine tissue follow-up procedures, paraffin blocks were embedded and taken in sections of 4-μm thickness from each block. The preparations for histopathological examination were stained with hematoxylin–eosin (HE) and examined with a light microscope (Olympus BX 51, Japan). The four‐point scoring system was used to evaluate the different stages of wound healing. Wound tissue sections were graded according to the following parameters: necrotic area, inflammatory cell infiltration, epithelialization, angiogenesis, keratinization, and collagen deposition. The above parameters were categorized as follows:

−: normal histology (no alterations);
+: < 25% (mild alterations);
++: 25%:50% (moderate);
+++: > 50% (severe).

### Immunohistochemical evaluation

For immunoperoxidase examination, tissue sections taken on adhesive (poly-L-Lysin) slides were deparaffinized and dehydrated. Then, endogenous peroxidase was inactivated by keeping in 3% H_2_O_2_ for 10 minutes. Then, the tissues were boiled in 1% antigen retrieval [citrate buffer (pH+6.1) 100X] solution and left to cool at room temperature. To prevent nonspecific background staining in the tissues, the sections were incubated with protein block for 5 minutes. Then, primary antibody (Tnf-α Cat No.: sc-52746, Dilution Ratio: 1/100, Santa Cruz Biotechnology, United States of America) was dropped onto the tissues and incubated according to the instructions for use. 3-3’ Diaminobenzidine (DAB) chromogen was used as chromogen in the tissues. Stained sections were examined with a light microscope assessed by ZEISS Zen Imaging Software (Zeiss Axio Germany)^16^.

### Double immunofluorescence evaluation

For immunoperoxidase examination, tissue sections taken on adhesive (poly-L-Lysin) slides were deparaffinized and dehydrated. Then, endogenous peroxidase was inactivated by keeping in 3% H_2_O_2_ for 10 minutes. Then, tissues were boiled in 1% antigen retrieval [citrate buffer (pH+6.1) 100X] solution and left to cool at room temperature. Sections were incubated with protein block for 5 minutes to prevent nonspecific background staining in tissues. Then, primary antibody (HSP70 Cat No.: FNab04048, Dilution Ratio: 1/100, Fine Test, United States of America) was dropped onto the tissues and incubated according to the instructions for use. Immunofluorescence secondary antibody was used as a secondary marker (FITC Cat No.: ab6785 Diluent Ratio = 1/1,000, Abcam) and kept in the dark for 45 minutes. Then, the second primary antibody (Col1 Cat No.: sc-293182, Dilution Ratio = 1/100, Santa Cruz Biotechnology, United States of America) was dropped on the tissues and incubated according to the instructions for use. Immunofluorescence secondary antibody was used as a secondary marker (Texas Red Cat No.: ab6719 Diluent Ratio: 1/1,000, Abcam, United Kingdom) and kept in the dark for 45 minutes. Then, DAPI with mounting medium (Cat No.: D1306 Dilution Ratio = 1/200, Thermo Fisher Scientific, United Kingdom) was dropped on the sections and kept in the dark for 5 minutes, and the sections were covered with a coverslip. The stained sections were examined under a fluorescence microscope and assessed by ZEISS Zen Imaging Software (Zeiss Axio Germany)^16^.

### Statistical analysis

The Statistical Package for the Social Sciences (SPSS) for Windows (Armonk, New York, IBM Corp.), version 13.0, was used for statistical analysis, and the data were evaluated with p < 0.05, considered significant. Duncan’s test was used for comparison between groups. The non-parametric Kruskal–Wallis’ test was used to detect group interaction, and the Mann–Whitney’s U test was used to determine differences between groups. To determine the intensity of positive staining from the images obtained as a result of immunohistochemical and immunofluorescence staining, five random areas were selected from each image and evaluated in the ZEISS Zen Imaging Software program. The data were statistically described as mean and standard deviation (mean ± SD) for % area. A one-way analysis of variance followed by Tukey’s test was performed to compare positive immunoreactive cells and immunopositive stained areas with healthy controls. For this test, *p* < 0.05 was considered significant, and the data were presented as mean ± SD.

## Results

### Body weight

**Figure 1:**
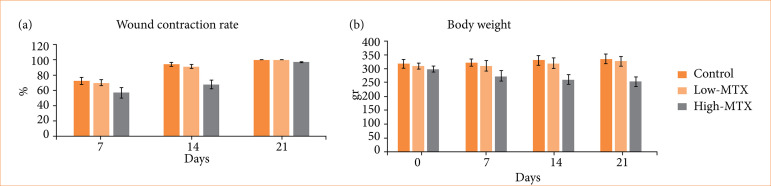
Changes compared to baseline in body weight and wound contraction rates in rats after treatment.

The post-treatment change in body weight compared to baseline (pre-treatment) in rats is presented in Fig. 1. A gain in body weight was observed in the control and low-MTX groups at all post-treatment dates compared to pre-treatment, whereas significant weight loss was recorded in the high-MTX group (*p* < 0.05).

MTX: methotrexate. Source: Elaborated by the authors.

### Wound contraction evaluation

**Figure 2:**
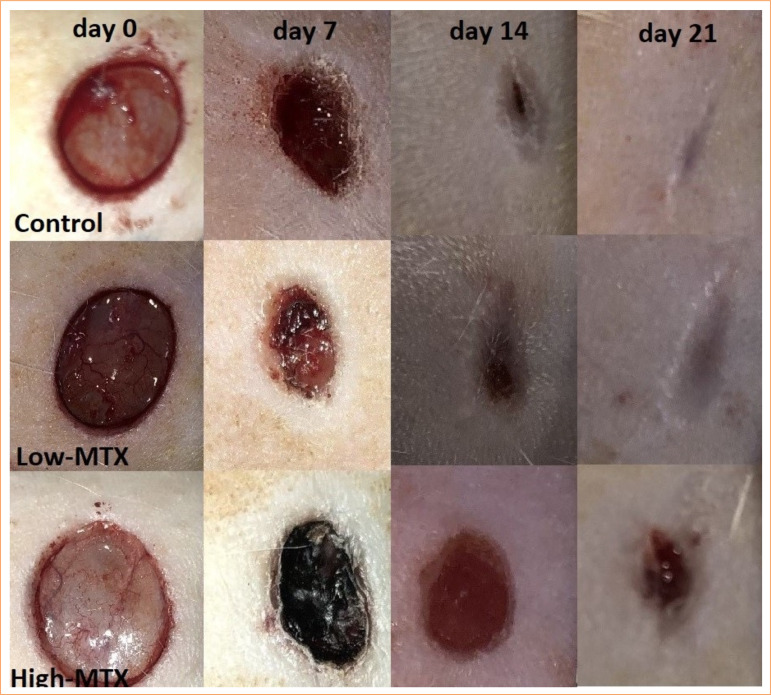
Morphological representation of rats wound showing various phases of wound healing.

The percentage of wound contraction was evaluated by considering the initial size of the wounds as 100% (Fig. 2). At seven days post-surgery, morphometric analyses revealed a larger wound closure area in the control group (72.66 ± 4.90%), followed by the low-MTX (70.01 ± 4.12%) and high-MTX groups (56.90 ± 6.82%). The contraction rate of MTX-treated wounds was lower than that of the control group. A statistically significant delay occurred in the high-MTX group compared to the other groups (*p* < 0.001). At 14 days post-surgery, a larger wound closure area was found in the control group (94.21 ± 2.97%), followed by the low-MTX (90.67 ± 2.26%) and high-MTX (67.57 ± 6.02%) groups. Significant differences were observed between groups (p < 0.05). At 21 days post-surgery, animals treated with saline or low-dose MTX had very similar scar tissue. In animals treated with high-dose MTX, the edges of the wound were narrowed, and most of the wound was closed, leaving a small open surface in the middle of the injury. Morphometric analyses revealed a larger wound closure area in the control and low-MTX groups (100.00 ± 0.00%), followed by the high-MTX group (97.16 ± 0.84%). Wound closure was significantly delayed in the high-MTX group compared to the other groups (*p* < 0.001). The wound contraction rates on days 7, 14, and 21 after injury are presented as percentages in Fig. 1.

MTX: methotrexate. Source: Elaborated by the authors.

### Biochemical evaluation

**Figure 3:**
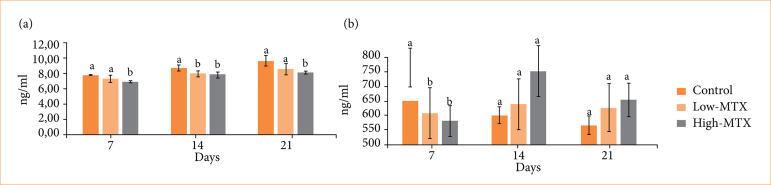
Wound tissue **(a)** hydroxyproline and **(b)** VEGF levels on days 7, 14, and 21.

MTX: methotrexate; VEGF: vascular endothelial growth factor. Source: Elaborated by the authors.

Wound tissue hydroxyproline and VEGF levels are shown in Fig. 3. The hydroxyproline levels in the high-MTX group were lower at all times compared to the other groups. Although the hydroxyproline levels in the low-MTX group were lower than those of the control group at all times, the difference was statistically significant only on day 14 (*p* < 0.05). The wound VEGF levels in the MTX-treated group were significantly lower than in the control group on day 7 (*p* < 0.05).

### Histopathological evaluation

**Figure 4:**
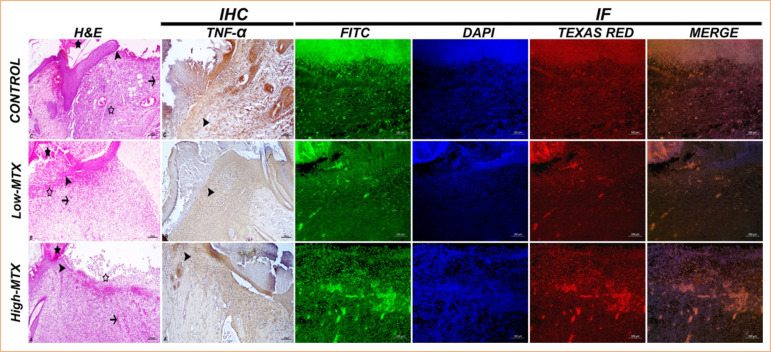
Day 7: skin tissue, H&E [epithelial regeneration (arrowhead), angiogenesis (arrow), necrotic mass (asterisk) and inflammation (empty asterisk)], IHC [TNF-α expressions-cytoplasmic TNF-α in inflammatory cells (arrowhead)], IHC-P [HSP70 expressions (FITC), Col1 expressions (Texas Red), IF]. Bar: 100 µm.

MTX: methotrexate; H&E: hematoxylin and eosin; TNF: tumor necrosis factor; FITC: fluorescein isothiocyanate; DAPI: 4’,6-diamidino-2-phenylindole; IHC: immunohistochemistry; IF: immunofluorescence. Source: Elaborated by the authors.

**Figure 5:**
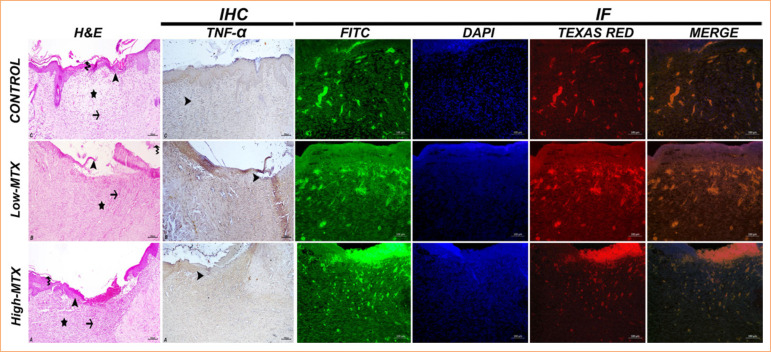
Day 14: skin tissue, H&E [epithelial regeneration (arrowhead), angiogenesis (arrow), tight collagenization (asterisk) and keratinization (curved arrow)], IHC [TNF-α expressions-cytoplasmic TNF-α in inflammatory cells (arrowhead)], IHC-P [HSP70 expressions (FITC), Col1 expressions (Texas Red), IF]. Bar: 100 µm.

MTX: methotrexate; H&E: hematoxylin and eosin; TNF: tumor necrosis factor; FITC: fluorescein isothiocyanate; DAPI: 4’,6-diamidino-2-phenylindole; IHC: immunohistochemistry; IF: immunofluorescence. Source: Elaborated by the authors.

**Figure 6:**
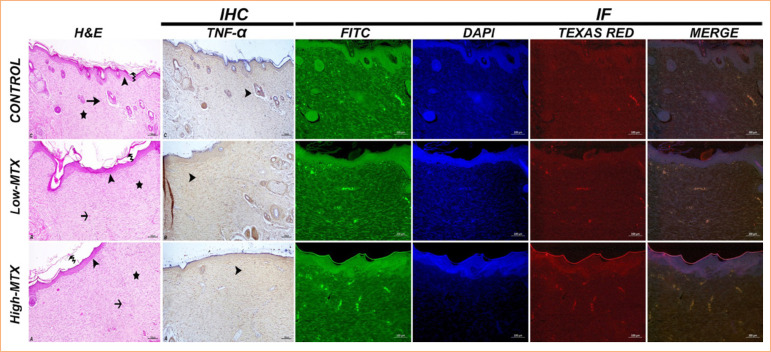
Day 21: skin tissue, H&E [epithelial regeneration (arrowhead), angiogenesis (arrow), loose collagenization (asterisk) and keratinization (curved arrow)], IHC [TNF-α expressions-cytoplasmic TNF-α in inflammatory cells (arrowhead)], IHC-P [HSP70 expressions (FITC), Col1 expressions (Texas Red), IF,], Bar:100µm.

MTX: methotrexate; H&E: hematoxylin and eosin; TNF: tumor necrosis factor; FITC: fluorescein isothiocyanate; DAPI: 4’,6-diamidino-2-phenylindole; IHC: immunohistochemistry; IF: immunofluorescence. Source: Elaborated by the authors.

The histopathological examination of skin samples showed a severe necrotic mass and exudation in the wound area in all groups on the seventh day. Severe inflammation was observed in the control group, moderate inflammation in the low-MTX group, and milder inflammation in the high-MTX group. Angiogenesis was significant in the control group and moderate in both MTX groups (Fig. 4). On the 14^th^ day, high epithelial regeneration in the wound area, moderate angiogenesis, significant tight collagenization, and moderate keratinization in the dermis were detected in the control group. In the low-MTX group, moderate epithelial regeneration, significant angiogenesis in the dermis, moderately tight collagenization, and mild keratinization were detected. In the high-MTX group, moderate epithelial regeneration, significant angiogenesis in the dermis, mildly tight collagenization, and mild keratinization were observed (Fig. 5). On the 21^st^ day, high-epithelial regeneration, loose collagenization, and keratinization were detected in the control group. In the low-MTX group, high-epithelial regeneration, moderate loose collagenization, and keratinization were detected. In the high-MTX group, moderate epithelial regeneration, keratinization, and mild loose collagenization were observed (Fig. 6). The histopathology scores in the experimental groups were lower than those of the control group, and the lowest score in the high-MTX group was statistically significant compared to the control group (*p* < 0.05) at all times. The histopathological scores are summarized in Table 1.

**Table 1:** Scoring of histopathological findings observed on days 7, 14 and 21.

Days	Histopathological Parameters	Control	Low-MTX	High-MTX
7^th^ day	Necrotic area	+++	+++	+++
	Inflammation	+++	++	+
	Angiogenesis	+++	++	++
14^th^ day	Epithelization	+++	++	++
	Keratinization	++	+	+
	Tight collagenization	+++	++	+
	Angiogenesis	++	+++	+++
21^st^ day	Epithelization	+++	+++	++
	Keratinization	+++	++	++
	Loose collagenization	+++	++	+
	Angiogenesis	+	++	+++

MTX: methotrexate;

+ mild;

++ moderate;

+++ severe.

Source: Elaborated by the authors.

### Immunohistochemical and immunofluorescent staining evaluation

In the immunohistochemical examinations performed on skin samples, on the seventh day, TNF-α expression was detected in the inflammatory cell cytoplasm and around the vessels in the dermis; it was high in the control group and moderate in the MTX groups (Fig. 2). While TNF-α expression decreased in the control group on the 14^th^ and 21^st^ days, it increased in the low-MTX group. In the high-MTX group, TNF-α expression decreased on day 14 and increased on day 21 (Figs. 5 and 6).

**Figure 7:**
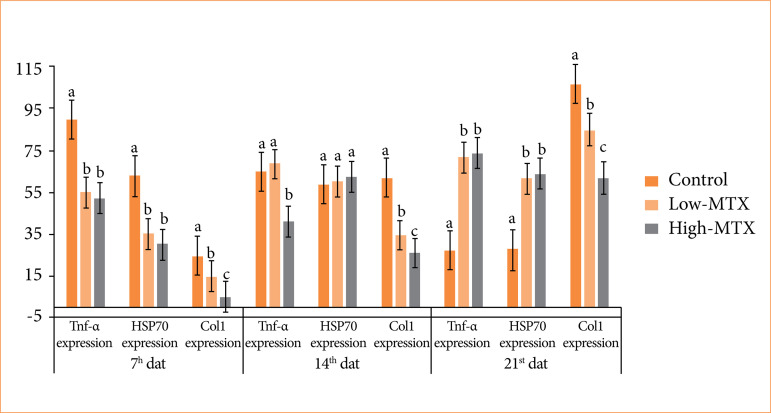
Data of immunohistochemical and immunofluorescence findings on days 7, 14 and 21.

MTX: methotrexate; TNF: tumor necrosis factor. Source: Elaborated by the authors.

The immunofluorescence staining performed on the skin samples showed that the control group had significant HSP70 expression compared to the MTX groups on day 7. In the control group, HSP70 expression trended downward from the 14^th^ to the 21^st^ day. In contrast, in the MTX groups, HSP70 expression trended gradually upward over the same period. At all times, collagen expression continued to increase in all groups. The degree of collagen expression was realized as control group, low-MTX group, and high-MTX group, respectively (Figs. 4, 5, and 6). The immunohistochemical and immunofluorescent expression statistical analysis data are presented in Fig. 7.

## Discussion

Wound healing is an orderly progression of carefully orchestrated, complex, interrelated processes that restore tissues’ anatomical and functional integrity. Any process, treatment, or intervention that alters or disrupts this progression can inhibit wound healing. Although chemotherapy drugs are intended to target rapidly dividing cancer cells, they can also affect other cell types. This often leads to the development of chronic wounds as cytotoxicity affects the skin cells and macrophages involved in wound healing^17^. MTX acts as an anticancer agent with strong antimetabolic and antiproliferative effects. In addition, MTX can have toxic effects on various organs, including the skin, and can significantly impede wound closure at various stages (inflammation, proliferation, and maturation).

There is limited evidence regarding MTX’s effect on wound healing. Enhancing our understanding of its effects on the wound-healing process will contribute to a better comprehension of the secondary long-term effects of MTX treatment^10^. As far as we know, no previous satisfactory study has shown the effect of MTX on excisional wound healing. Therefore, we investigated the effects of both low- and high-doses of MTX on wound healing via morphometric, biochemical, histopathological, and immunohistochemical means in an excisional wound model in rats. We used the low- and high-dose model (assessed on days 0, 7, 14, and 21) used by Cavalcanti et al.11 for fracture healing in rats. MTX is used as an antineoplastic drug in high doses and as an immunosuppressive and anti-inflammatory drug in low doses.

We also recorded the body weights of the rats before and after wounding. All rats were fed *ad libitum* throughout the experiment. In high-MTX rats, decreased food intake and body weight were noted on days 7, 14, and 21 compared to pre-treatment (*p* < 0.05). Conversely, weight gain was detected in rats treated with low-dose MTX and saline. MTX exerts its primary toxic effects against the rapidly replicating cells of the bone marrow and gastrointestinal epithelium. In previous studies, high-dose MTX administration in rats resulted in adverse effects such as weight loss, diarrhea, and nose bleeding due to gastrointestinal toxicity^18^. In this study, the weight loss in high-dose MTX-treated rats might reflect direct gastrointestinal toxicity or their reduced feed and water intake.

Contraction, or a reduction in wound size, and epithelialization are two crucial stages in the wound-healing process. Skin wound contraction is the result of fibroblast differentiation into myofibroblasts, and epithelialization is the result of keratinocyte migration and proliferation. Several reports have shown that fibroblasts and keratinocytes interact and coordinate the wound-healing process, although it involves different phenomena performed by different cell types^19^. In a recent study investigating the effects of a single 30 mg/kg dose, a single 200 mg/kg dose, and repeated 1.5 mg/kg doses every three days on wound healing in excisional wounds in mice, the wounds closed by contraction on the 10^th^ day in the control group and on the 14^th^ day in the MTX groups. MTX applied repeatedly and in high doses caused a significant delay in wound closure compared to the control group^10^. We determined that there was a significant delay in wound contraction in the high-MTX group compared to the other groups at all times (*p* < 0.001). In the low-MTX group, a significant delay in the wound contraction rate was observed only on the 14^th^ day compared to the control group (*p* < 0.05). The delayed contraction in MTX-treated wounds aligns with the findings of recent studies. MTX has been demonstrated to reduce fibroblast proliferation during the wound-healing process. Consequently, the reduced transformation of fibroblasts to myofibroblasts may be one of the reasons for delayed wound contraction. These results support the findings of studies on MTX’s antiproliferative and antifibrotic properties^10^.

The inflammatory phase, in which macrophages activate and key cytokines and growth factors are released, is responsible for protecting the wound against infection or debridement. This phase is, therefore, crucial for the correct progression of wound healing. However, when the inflammatory phase is interfered with, the healing process may be affected, and wound healing may be impaired20. MTX, which has anti-inflammatory effects, inhibits the enzyme 5-aminoimidazole-4-carboxamide ribonucleotide transformylase, which is involved in purine synthesis, and increases adenosine, which has anti-inflammatory effects. As a result of the increased adenosine inside and outside of cells, the release of proinflammatory cytokines and neutrophil, macrophage, and T-cell migration are inhibited.

Increased adenosine levels have been shown to mediate MTX’s anti-inflammatory effects in several in-vitro and in-vivo studies^21^. In a study investigating the effects of MTX on dental pulp by administering 0.25 mg/kg intraperitoneally three times a week in rats, MTX reportedly acted as an anti-inflammatory agent by suppressing polymorphonuclear and mononuclear cells and reducing the immunological response^22^. We found that inflammation was suppressed in rats treated with MTX, and the effect was stronger in the high-MTX group during the early period of wound healing compared to the control group. These data suggest that MTX exerts a dose-dependent anti-inflammatory effect and support its anti-inflammatory effects, which have been demonstrated in many studies^10^^,^ 21 ^,^ 23.

The proliferative phase of wound healing is characterized by macrophages’ secretion of TNF-α, which, in turn, induces proteoglycan and fibronectin generation by fibroblasts to facilitate extracellular matrix formation in wounded tissues. The ability of MTX to suppress lymphocyte (T and B) proliferation and function, macrophage stimulation, chemotaxis, and histamine release from basophils may be related to its reduction of TNF-α levels^24^. In one study, a decrease in TNF-α levels in brain tissue was observed in rats treated with MTX at doses of 5 and 10 mg/kg intraperitoneally for a short time (four days) compared to a control group. MTX is known to suppress TNF-α synthesis in monocytes, macrophages, and T-cells, as well as the TNF-induced activation of NF-κβ, and it suppresses TNF-α activity in vitro, possibly through a mechanism related to adenosine release^25^. Neurath et al.^26^ reported that MTX had a regulatory effect on *in-vitro*, *in-vivo*, and *ex-vivo* cytokine production in mice, significantly suppressing TNF-α production. They stated that the effect of MTX on TNF-α production was partially dependent on folate. As expected from its widely known anti-inflammatory and immunosuppressive effects, our results showed that TNF-α expression was suppressed in both MTX-treated groups in the early phase of wound healing. This is consistent with the results of previous studies^24^^–^ 26. Our findings suggested that the suppression of TNF-α expression may delay wound healing by interfering with fibroblast proliferation and these cells’ formation of the extracellular matrix.

Heat shock proteins (HSPs) are crucial factors for the wound-healing process due to their roles in cell proliferation, collagen synthesis, the modulation of inflammation, and the clearance of wound debris. HSP expression in cells is managed at baseline levels under normal physiological conditions. Under wound-healing stress, HSP expression increases dramatically to accelerate wound healing. Therefore, any disruption in the expression and function of HSPs in response to various cellular stresses can cause various wound-healing complexities^27^. A previous study revealed that TNF-α, one of the pro-inflammatory cytokines, induces HSP70 in the *in vitro*-medium^28^. We assumed that the suppression of HSPs in the early period in the MTX groups may have reduced the presence of HSP70 in the environment by indirectly suppressing TNF-α, a pro-inflammatory cytokine, in the early period of wound healing, although MTX does not exert a direct effect on HSPs.

VEGF contributes to wound healing by promoting angiogenesis, collagen production, granulation tissue formation, and epithelialization. VEGF expression increases in skin wounds, reaching a maximum seven days after injury. Researchers have shown that MTX suppresses inflammatory cells in human nasal organ cultures and also suppresses VEGF by reducing the activity of angiopoietin-1, a positive regulator of angiogenesis, and increasing the activity of angiopoietin-2, an inhibitor^29^. In a previous study using a model of corneal fibroblast growth factor-induced neovascularization in rabbits, topically applied MTX inhibited angiogenesis in the rabbits’ corneas. The authors hypothesized that MTX’s antiangiogenic mechanism might reflect its inhibition of both macrophage invasion during early angiogenesis and endothelial cell proliferation^30^. Oliveira et al.^31^ reported that the controlled release of MTX from polycaprolactone implantable devices showed a clear antiangiogenic effect in a mouse model of inflammation and angiogenesis. They revealed that this antiangiogenic mechanism was also associated with VEGF downregulation and the inhibition of TNF-α, a potent angiogenic factor. We found that angiogenesis was suppressed in parallel with VEGF in rats treated with MTX during the early stages of wound healing, compared to untreated rats. The effect was especially significant in rats treated with high-dose MTX compared to untreated rats (*p* < 0.05). These results are consistent with the results of previous studies^29^-^31^. We assume that MTX has an antiangiogenic effect as a result of downregulating VEGF levels and suppressing the expression of TNF-α, which is a strong angiogenic factor.

Collagen is the main component of the ECM that is synthesized by fibroblasts, and it plays an important role in all stages of wound healing, including hemostasis, inflammation, proliferation, and remodeling. It primarily orchestrates the orderly structuring and aggregation of recently generated fibers and granulation tissue in the wound bed, thereby establishing an environment that is conducive to effective tissue regeneration. Collagen, the main structural component of granulation tissue, contains the amino acid proline. Hydroxyproline is a biochemical marker for collagen tissue and a positive indicator of healing progress^32^.

Matrix metalloproteases (MMPs) are involved in the degradation of collagen and other ECM components. MMPs have been shown to modulate skin regeneration and hypertrophic scarring in several studies. In patients with extensive hypertrophic scarring after burns, MMP-1 protein is highly expressed^33^. MTX increases MMP-1 expression by dermal fibroblasts. It is believed that MTX has the potential to become a novel therapeutic option for hard-to-treat fibroproliferative disorders such as hypertrophic scarring and keloids^34^.

Some early studies have highlighted that, in the early stages of wound healing, MTX causes a significant delay in wound fibroplasia with a transient decrease in the tensile strength of the wound, depending on the dose^9^. Kolzet et al.^22^ stated that MTX given systemically (intraperitoneally) in rats may delay the repair of pulp tissue by interfering with fibroblast activation. Medellín-Luna et al.^10^ noted that MTX treatment of excisional wounds in mice severely impaired collagen synthesis and deposition, which are indicators of wound maturation and tissue tensile strength. Meanwhile, Nabai et al.^34^ reported that MTX caused a significant decrease in fibroblast cell proliferation, metabolic activity, and type-1 collagen levels in primary human dermal adult and neonatal fibroblast cell cultures treated with MTX *in vitro*. MTX not only inhibits fibroblasts’ mitogenic potential but can also cause apoptosis. MTX reduced the secretion of type-I collagen and increased the production of MMP-1 and sGAG. The observation that MTX downregulates the activity of dickkopf-1 (DKK1) and TAGLN may partly explain these results. MTX applied locally to the laminectomy area at different doses in rats decreased the number of fibroblasts, depending on the dose and the density of collagen independent of the dose, compared to saline treatment. At the same time, the tissue hydroxyproline levels in rats treated with MTX were significantly reduced compared to those treated with saline. The same study showed that MTX causes apoptotic cell death in fibroblasts by inducing endoplasmic reticulum stress in fibroblast cells cultured from the epidural scar tissue of rats^35^. We examined both hydroxyproline levels and collagen expression in the tissue and observed that MTX dose-dependently decreased hydroxyproline levels in wounds and suppressed collagen deposition in the wound bed. These results are consistent with the results of previous *in-vivo* and *in-vitro* studies^10^^,^ 35 ^,^ 36. Our results suggested that MTX may inhibit both the proliferation and activation of fibroblasts, which are responsible for collagen synthesis, thereby decreasing collagen synthesis and decreasing hydroxyproline levels in the wound tissue.

Keratinocytes are essential to wound healing. They are responsible for the rapid formation of an epithelium over damaged tissue, establishing a barrier between the internal environment and the external environment. These cells exhibit multifaceted functionality, producing a variety of factors to foster re-epithelialization. In addition, keratinocytes actively generate mediators that not only promote angiogenesis but also augment the synthesis of connective tissue matrix, thereby exerting a constructive influence on connective tissue repair mechanisms^37^. The skin biopsy samples of patients treated with high-dose MTX showed multiple keratinocyte dysfunctions, such as impaired keratinocyte maturation, widening intercellular spaces, irregular large nuclei, and apoptosis^38^. MTX exerts a dose-dependent cytotoxic effect in human keratinocytes.

Cell death has been associated with the upregulation of pro-apoptotic markers (Bax and Bad). In addition, in the vascular plexus, MTX caused irritant effects that translated into hemorrhage, lysis, and vascular stasis. MTX’s negative effect on keratinocytes directly affects epithelialization, because keratinocytes are the executors of the re-epithelialization process due to their migration and proliferation properties^39^.

Ahmed et al.^40^ reported that rats treated with 20-mg/kg intraperitoneally MTX had histopathological significant thickening of the oral and lingual mucosal epithelium and showed more damage to basal cells and connective tissue compared to untreated rats. In an *in-vitro* study investigating the effects of different concentrations of MTX on the proliferation of human immortalized keratinocytes (HaCaT), MTX showed antiproliferative effects and induced apoptosis by arresting cells in the G0/G1 phase of the cell cycle. In the same study, a statistically significant decrease in the thickness of the stratum basale and stratum corneum of the epidermis and smooth muscle tissue was found in wounds treated with high and repeated doses of MTX in a mouse excisional wound model compared to the control group^39^. In our study, delayed epithelialization and keratinization occurred in wounds treated with both low- and high-dose MTX compared to the control group. In the high-MTX group, the delay in epithelialization continued at 21 days. These results align with previous studies^10^^,^ 40. Keratinocytes are among the most mitotically active cells in the body. The delay in epithelialization on day 21 in the high-MTX group compared to the other groups may indicate that keratinocytes are the most sensitive cells in the wound-healing process to MTX side effects^38^.

## Conclusion

MTX therapy is a frequently preferred drug in the treatment of rheumatic diseases and cancer. Wound healing was impaired, especially in rats treated with high-dose MTX. We suggest that MTX’s dose-dependent adverse effects on wound healing may be due to its antiproliferative, antifibrotic, anti-inflammatory, and antiangiogenic effects. As a result of studies on MTX, postoperative MTX use should be carefully evaluated, and a better understanding of the effects of MTX on wound healing will lead to a better understanding of the long-term effects of MTX treatment.

## Data Availability

All data sets were generated or analyzed in the current study.
